# YB‐1 regulates mesothelioma cell migration via snail but not EGFR, MMP1, EPHA5 or PARK2

**DOI:** 10.1002/1878-0261.13367

**Published:** 2023-01-25

**Authors:** Karin Schelch, Sebastian Eder, Benjamin Zitta, Monica Phimmachanh, Thomas G. Johnson, Dominik Emminger, Andrea Wenninger‐Weinzierl, Caterina Sturtzel, Hugo Poplimont, Alexander Ries, Konrad Hoetzenecker, Mir A. Hoda, Walter Berger, Martin Distel, Balazs Dome, Glen Reid, Michael Grusch

**Affiliations:** ^1^ Center for Cancer Research and Comprehensive Cancer Center Medical University of Vienna Austria; ^2^ Department of Thoracic Surgery Medical University of Vienna Austria; ^3^ University of Technology Sydney NSW Australia; ^4^ The University of Sydney NSW Australia; ^5^ St. Anna Children's Cancer Research Institute, Innovative Cancer Models Vienna Austria; ^6^ National Koranyi Institute of Pulmonology Budapest Hungary; ^7^ Department of Thoracic Surgery Semmelweis University and National Institute of Oncology Budapest Hungary; ^8^ Department of Pathology Dunedin School of Medicine New Zealand; ^9^ The Maurice Wilkins Centre University of Otago Dunedin New Zealand

**Keywords:** cell migration, EGFR, pleural mesothelioma, snail, YB‐1

## Abstract

Pleural mesothelioma (PM) is characterized by rapid growth, local invasion, and limited therapeutic options. The multifunctional oncoprotein Y‐box‐binding protein‐1 (YB‐1) is frequently overexpressed in cancer and its inhibition reduces aggressive behavior in multiple tumor types. Here, we investigated the effects of YB‐1 on target gene regulation and PM cell behavior. Whereas siRNA‐mediated *YB‐1* knockdown reduced cell motility, *YB‐1* overexpression resulted in scattering, increased migration, and intravasation *in vitro*. Furthermore, YB‐1 stimulated PM cell spreading in zebrafish. Combined knockdown and inducible overexpression of *YB‐1* allowed bidirectional control and rescue of cell migration, the pattern of which was closely followed by the mRNA and protein levels of EGFR and the protein level of snail, whereas the mRNA levels of *MMP1*, *EPHA5*, and *PARK2* showed partial regulation by YB‐1. Finally, we identified snail as a critical regulator of YB‐1‐mediated cell motility in PM. This study provides insights into the mechanism underlying the aggressive nature of PM and highlights the important role of YB‐1 in this cancer. In this context, we found that YB‐1 closely regulates EGFR and snail, and, moreover, that YB‐1‐induced cell migration depends on snail.

## Graphical abstract

The oncoprotein YB‐1 controls aggressive behavior of pleural mesotheloma cells by regulating the expression of Snail. Knockdown of YB‐1 via siRNA resulted in a decrease, while doxycycline‐induced YB‐1 overexpression strongly induced cell scattering, migration and invasion. Modulated YB‐1 levels directly correlate with, and the respective phenotypes depend on, Snail expression.

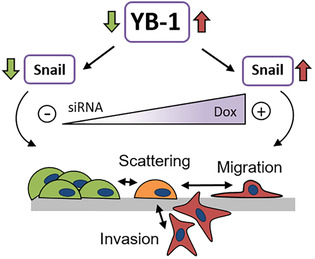

**Abbreviations**
ATCC: American Type Culture Collection
CCID: circular chemorepellent‐induced defect
CDS: coding sequence
EGFR: epidermal growth factor receptor
GFP: green fluorescence protein
PM: pleural mesothelioma
RFP: red fluorescent protein
UTR: untranslated region
YB‐1: Y‐box‐binding protein 1

## 1 Introduction

Pleural mesothelioma (PM) is an aggressive, mainly asbestos‐induced malignancy arising from the mesothelial linings of the pleural cavity. Despite limitations on asbestos use in many countries of the world, the global burden from asbestos‐related diseases including PM will remain high for many years [1]. The standard of care treatment for PM patients involves chemotherapy with a platinum drug plus pemetrexed which is combined with surgery and radiotherapy in selected cases [2]. More recently, combination immunotherapy with nivolumab plus ipilimumab has been added to the list of available treatments. While this provides a significant improvement over standard of care chemotherapy (overall survival 18.1 months vs 14.1 months), the objective response rate was only 40% and the overall prognosis still remains poor [3]. Importantly, local tumor spread along the pleural surface and invasion into surrounding tissues remain major challenges in PM treatment and often cause rapid tumor recurrence even after multimodality therapy [4].

YB‐1 is a multifunctional nucleic acid‐binding oncoprotein that is frequently overexpressed in tumors, involved in all hallmarks of cancer, and has been described to confer growth‐ and metastasis‐promoting capabilities to multiple types of cancer cells [5, 6]. YB‐1 has been shown to mediate its multiple functions through the regulation of a variety of target genes including both transcriptional and post‐transcriptional effects [5, 6]. Regulation of the epidermal growth factor receptor (EGFR), for instance, depended on the binding of YB‐1 to specific regions in the EGFR promoter [7, 8]. In contrast, YB‐1 was able to activate cap‐independent translation of mRNAs encoding snail, hypoxia‐inducible factor (HIF1α) and other transcription factors without affecting their mRNA expression [9]. In PM, we have previously shown that YB‐1 overexpression is due, at least in part, to loss of its negative regulator microRNA miR‐137 [10]. Moreover, RNA‐interference‐mediated knockdown of YB‐1 significantly reduced cell growth, migration and invasion in PM cells [10]. However, the downstream target genes of YB‐1 that mediate these effects are yet to be defined in the context of PM.

In the current study, we further explore the role of YB‐1 in PM and demonstrate that YB‐1 overexpression causes cell scattering and enhances cell migration *in vitro*, increases intravasation in a 3D co‐culture model, and stimulates cell spreading in a zebrafish xenotransplantation model. Furthermore, we investigate the role of several previously described and novel targets of YB‐1 and identify snail as a critical regulator of YB‐1‐mediated cell motility in PM.

## 2 Materials and methods

### 2.1 Cell culture

All cell lines used in this study were either purchased from ATCC (MSTO‐211H, HEK‐293, Met‐5A), provided by collaboration partners (MM05, Ren, SPC212, BEC, LEC), or established at the Medical University of Vienna (VMC23, VMC40, Meso62, and Meso84) as recently described [11], and authenticated by array comparative genomic hybridization and STR profiling within the past three years. Met‐5A is an immortalized, nonmalignant mesothelial cell line. Ren and VMC23 were established from epithelioid, Meso62, Meso84 from sarcomatoid and the other PM cell lines from biphasic PM. All PM and mesothelial cells were cultivated in RPMI medium supplemented with 10% heat‐inactivated fetal bovine serum (FBS) in a humidified atmosphere (37 °C, 5% CO_2_) and regularly checked for *Mycoplasma* contamination. Immortalized endothelial cells (BEC, LEC) were grown in Endothelial Cell Growth Media (Lonza Group AG, Basel, Switzerland).

### 2.2 Transgenic cell lines

SPC212 cell lines stably and constitutively overexpressing YB‐1 (SPC212^YB1‐s^), and the respective vector controls (SPC212^VC‐s^) were generated by transfection with the expression constructs pIRES‐EGFP‐YB‐1 and pIRES‐EGFP [12], respectively, using Lipofectamine 3000 (Thermo Fisher Scientific, Waltham, MA, USA) and stable clones were established under constant selection pressure.

To establish the doxycycline‐inducible YB‐1 or RFP overexpression models, cells were infected with retroviral particles produced in HEK‐293 cells by co‐transfecting the retroviral expression plasmids pRXTOP‐YB‐1 and pRXTOP‐RFP with the helper plasmids VSV‐G and pgag‐pol‐gpt. The plasmids pRXTOP‐YB‐1 and pRXTOP‐RFP were generated by PCR amplification of the full open reading frames of YB‐1 and turboRFP, respectively, and subcloning the fragments into the doxycycline‐inducible retroviral expression plasmid pRetroX‐TetOne‐puro (Takara Bio, Kusatsu, Japan) with the In‐Fusion cloning kit (Takara Bio). Selection was performed in the absence of doxycycline. SPC212^YB1‐i, RFP^ were generated by infecting SPC212^YB1‐i^ with a second retrovirus using the constitutive expression plasmid pQCXIN‐RFP. SPC212^mCherry^ were generated by retroviral infection using the constitutive expression plasmid pQCXIP‐mCherry.

### 2.3 Transfection with siRNAs

The *YBX1*‐specific siRNAs si‐YB1^CDS^ (UUUGCUGGUAAUUGCGUGGAGGACC), si‐YB1^UTR^ (UAUUUCUUCUUGUUGGAUGACUAAA) and nonsilencing control siRNA (AAGCAACUUGGUAAGACUCGUGUGG) were purchased from GenePharma (Shanghai, China) and have been previously validated [10, 13]. The siRNAs targeting *EGFR* and *SNAI1* were obtained from Santa Cruz Biotechnology (Dallas, TX, USA). Cells were reverse transfected with 0.1% Lipofectamine RNAiMAX as described [10].

### 2.4 Drug treatment

Doxycycline (Selleckchem, Houston, TX, USA) was dissolved in PBS and used at a concentration of 100 ng·mL^−1^ in all *in vitro* experiments and 100 μg·mL^−1^ in the *in vivo* setting. The EGFR inhibitor erlotinib (Selleckchem) was dissolved in DMSO and used at a concentration of 10 μm.

### 2.5 Growth assay

Cells (2 × 10^3^ per well) were seeded into 96‐well plates. Where indicated, doxycycline (100 ng·mL^−1^) was added on the next day. After the indicated time points, the assay was stopped by freezing at −80 °C, and the cell proliferation was analyzed using a lysis buffer containing SYBR green as previously described [14].

### 2.6 Colony formation assay

Cells (2 × 10^3^) were seeded into 6‐well plates and on the next day treated as indicated. After 7–14 days, cells were fixed by methanol/acetic acid (3 : 1), stained with 0.1% crystal violet and imaged using a Zeiss Axiovert microscope (Carl Zeiss GmbH, Oberkochen, Germany). Afterward, clones were destained in 2% SDS and absorbance was measured at 560 nm. The distance to the nearest neighbor was determined using imagej (National Institute of Health, Bethesda, MD, USA).

### 2.7 Live‐cell imaging, migration and cell fate analysis

Live cell videos were generated using an EVOS FL Auto Live Cell Imager (Thermo Fisher Scientific), a Nikon Visitron Live Cell System (Visitron Systems GmbH, Puchheim, Germany) or an IncuCyte S3 Live‐Cell Analysis System (Sartorius AG, Göttingen, Germany).

Migration of single cells was manually tracked using imagej as previously described [13, 15]. Specifically, cells (3 × 10^3^) were seeded into 48‐well plates and, where indicated, treated with 100 ng·mL^−1^ doxycycline or 10 μm erlotinib. On the next day, videos were started using 30 min intervals. For knockdown experiments, cells were transfected with siRNA 1 day before seeding. For further analysis of migratory behavior including speed, mean square displacement (MSD), and origin plots, the DiPer migration tool for Microsoft Excel was used [16].

For the scratch assay, cells were grown into a confluent layer in a 24‐well plate and treated as indicated. A scratch was generated using a P200 pipet tip and its closure was monitored by videomicroscopy at the indicated time points. Migrated distance or % of scratch closure were manually measured using imagej.

Cell fate maps were manually generated from live‐cell videos with pictures taken every 10 min as previously described [17].

### 2.8 Gap assay

Cells (3 × 10^3^) were seeded in medium containing 3.2 mg·mL^−1^ methyl cellulose (Sigma‐Aldrich, St. Louis, MO, USA) into 96‐well suspension plates and allowed to form spheroids with or without doxycycline for 48 h. In parallel, GFP‐expressing LEC or BEC cells (1.8 × 10^4^) were seeded into 24‐well plates to form a confluent layer. After 2 days, spheroids were harvested, washed and placed on the endothelial cell layer. Pictures were taken on a Nikon Eclipse Ti300 microscope (Nikon, Tokyo, Japan) after 2 and 4 h with the bright field and GFP channel to visualize spheroids and endothelial cells, respectively. Areas of the spheroids and respective gaps were calculated using imagej.

### 2.9 Zebrafish model

Zebrafish (*Danio rerio*) mutants (*mitfa* ^*b692/b692*^; *ednrba* ^*b140/b140*^; *Tg(fli1a:EGFP)* ^*y1*^ *)* were maintained at standard conditions under license GZ:565304‐2014‐6 of the local authorities (Magistratsabteilung 58 of the City of Vienna) at CCRI [18, 19]. Zebrafish were bred at the CCRI zebrafish facility. The origin of transgenic and mutant strains can be obtained from the allele designation number through zfin.org. Tg(Fli1a:EGFP)y1 was originally established in the Weinstein lab. Ednrba^−/−^ b140 derive from the Kimmel lab and mitfa^−/−^ b692 from the Raible lab. Xenotransplantation of zebrafish embryos (license GZ:333989‐2020‐4) was performed as previously described [20].

Around 200–300 SPC212 cells were injected into the perivitelline space (PVS) of the zebrafish larvae at 2 days post fertilization (dpf). Xenotransplanted larvae were kept at 34 °C and sorted for larvae with tumor cells only in the PVS at 2 h post injection (hpi). Half of the selected embryos were incubated in E3 embryo medium supplemented with 100 μg·mL^−1^ doxycycline (Sigma‐Aldrich).

Automated imaging of xenografted zebrafish was carried out as described by Grissenberger et al. [20] using an Operetta CLS high‐content imager [PerkinElmer, Waltham, MA, USA; 5× air objective, brightfield (40 ms, 10%), GFP (excitation: 460–490 nm at 100%, emission: 500–550 nm for 400 ms), tagRFP (excitation: 530–560 nm at 100%, emission: 570/650 nm for 400 ms), 23 planes with a distance of 25 μm]. Pictures were taken 1 and 2 days post injection (dpi), and cells present in the tail were manually counted using imagej. To quantify extravascular cells, masks of vessels and tumor cells were generated in each respective fluorescent channel and subtracted using Fiji. Remaining cells were considered extravascular. A threshold of 20% was used to define minimal extravasation (< 20% outside the vessels) and extravasation (> 20% outside the vessels).

### 2.10 RNA isolation and qPCR

Total RNA was isolated from tumor cells or zebrafish larvae using the InnuPrep RNA Kit (Analytik Jena, Jena, Germany) and reverse transcribed with M‐MLV reverse transcriptase (Thermo Fisher Scientific). Quantitative real‐time PCR was performed on a CFX96 Thermocycler (BioRad, Hercules, CA, USA) using iTaq Universal Probes or SYBR Green Supermix (BioRad). Taqman probe IDs and primer sequences are listed in Table S1. Changes in gene expression are shown as log2 of $2^{-\Delta \Delta C_{t}}$ compared to the respective untreated control and are normalized to a reference gene as indicated.

### 2.11 Protein isolation and immunoblots

After transfection or treatment at the indicated concentrations and time points, cells were harvested in lysis buffer (150 mm NaCl, 50 mm HEPES, 10% glycerol, 1 mm EDTA, 0.5 mm Na_3_VO_4_, 10 mm NaF, 1% Triton X100, 1.5 mm MgCl_2_). Protein concentrations were determined using the BCA Protein assay (BioRad). Proteins were separated by SDS/PAGE and blotted onto PVDF membranes, and immunodetection was performed with the Clarity Western ECL Substrate kit (BioRad) using the following antibodies: YB‐1 (ab12148, Abcam, Cambridge, UK, 1 : 1000); EGFR (sc‐03‐G, Santa Cruz Biotechnology, 1 : 1000); snail (C15D3, Cell Signaling Technology, 1 : 1000); and Beta‐actin (A5441, Sigma, 1 : 2000). Secondary antibodies were purchased from Dako and used at a dilution of 1 : 10 000.

### 2.12 Statistical analysis

Unless stated otherwise, data were analyzed using graphpad prism 8 (GrapPad Software, San Diego, CA, USA) and are shown as means ± SEM of at least three independent experiments, each performed in triplicate. Differences were evaluated by Student's *T*‐test or ANOVA for comparisons of two or multiple groups, respectively, and considered statistically significant at *P* < 0.05.

## 3 Results

### 3.1 YB‐1 overexpression induces cell growth, colony formation, and scattering

**Fig. 1:**
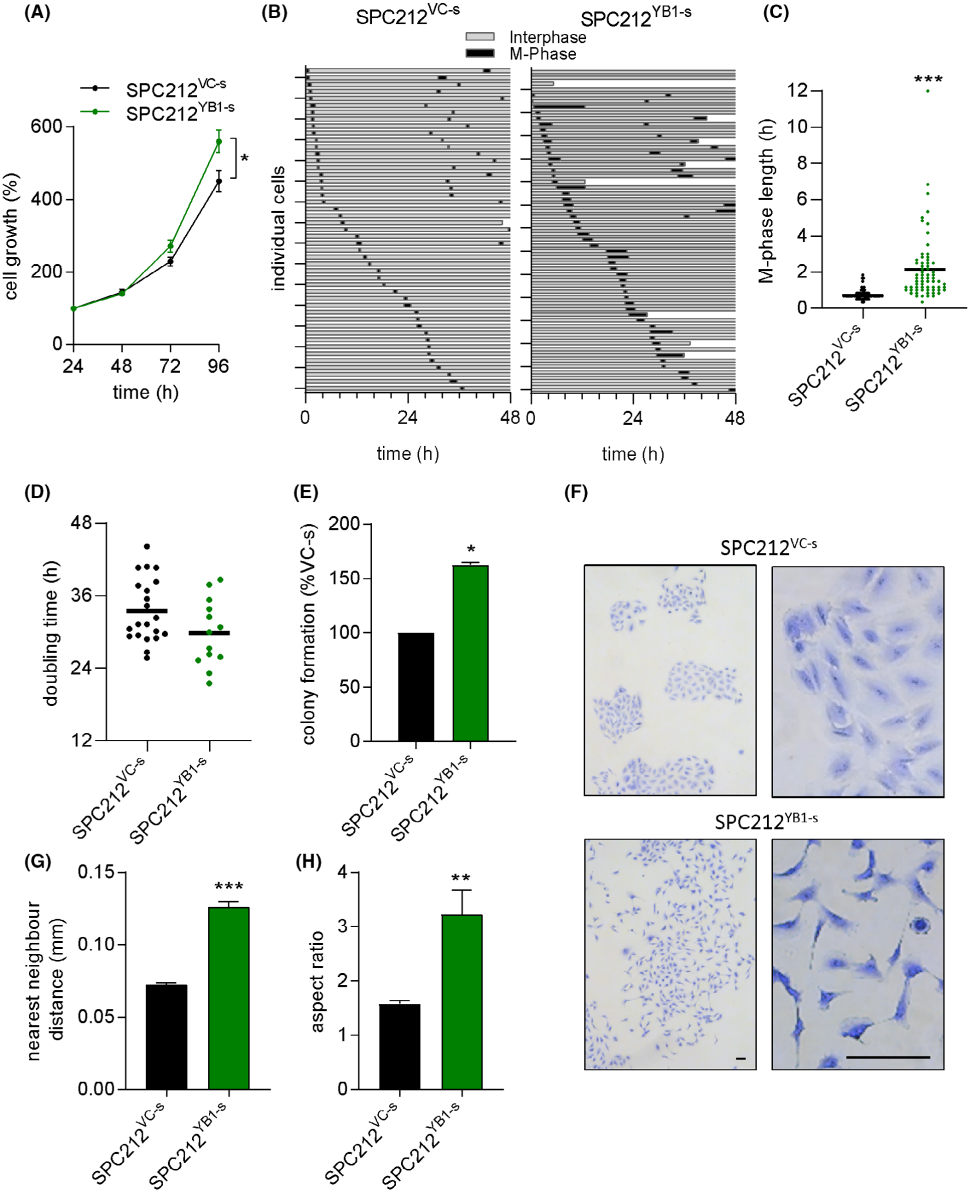
YB‐1 overexpression induces cell cycle alterations, scattering and morphology changes. (A) Cell growth of SPC212 cells stably overexpressing YB‐1 (SPC212^YB1‐s^) or the empty vector (SPC212^VC‐s^) after 96 h. Data are shown as mean ± SEM of 3 biological replicates performed in triplicate. (B) Cells were subjected to videomicroscopy over 48 h with 10 min intervals. Cell fate maps indicate interphase (gray) and M‐phase duration (black). Cells that died over the 48‐h period are indicated by the bar not reaching the end of the graph. Each bar represents one single cell. (C) M‐phase lengths and (D) doubling times of single cells from videos in (B). Each dot represents one single cell. The horizontal lines indicate the mean. (E) Quantification of colony formation assays. (F) Representative pictures of colony formation assays. Scale bar: 100 μm. (G) Distance to the nearest neighbor of > 160 cells and (H) aspect ratio of > 20 single cells, derived from colony formation assays. Bars show mean ± SEM. Student's *T*‐test, *P < 0.05, **P < 0.01, ***P < 0.001.

We previously demonstrated the overexpression of YB‐1 and its relevance for malignant growth in PM [10]. To further investigate the functions and targets of YB‐1 in PM, we generated a PM cell line stably overexpressing YB‐1 (SPC212^YB1‐s^) and a respective vector control (SPC212^VC‐s^), achieving overexpression of 2.3‐fold on mRNA and 1.5‐fold on protein level (Fig. S1). To analyze the impact of YB‐1 overexpression in this cell model, we first determined the effect on cell growth and found a moderate increase (Fig. 1A). For further characterization, we performed cell fate analysis of individual cells by videomicroscopy. Despite the higher number of cell deaths (indicated by the end of a bar before the 48 h time point) and an increase in M‐phase length, a tendency toward shorter doubling times in the YB‐1 overexpressing cells was seen, which is in accordance with enhanced cell growth (Fig. 1B–D). As a next step, we performed colony formation assays and found significantly increased colony formation in YB‐1 overexpressing cells compared to the control (Fig. 1E). Interestingly, colonies also showed a dramatic difference in growth pattern, with SPC212^YB1‐s^ exhibiting cell scattering and more elongated cell shapes, quantified by nearest neighbor distance and aspect ratio analysis, respectively (Fig. 1F–H).

### 3.2 YB‐1 overexpression leads to strongly enhanced cell migration

**Fig. 2:**
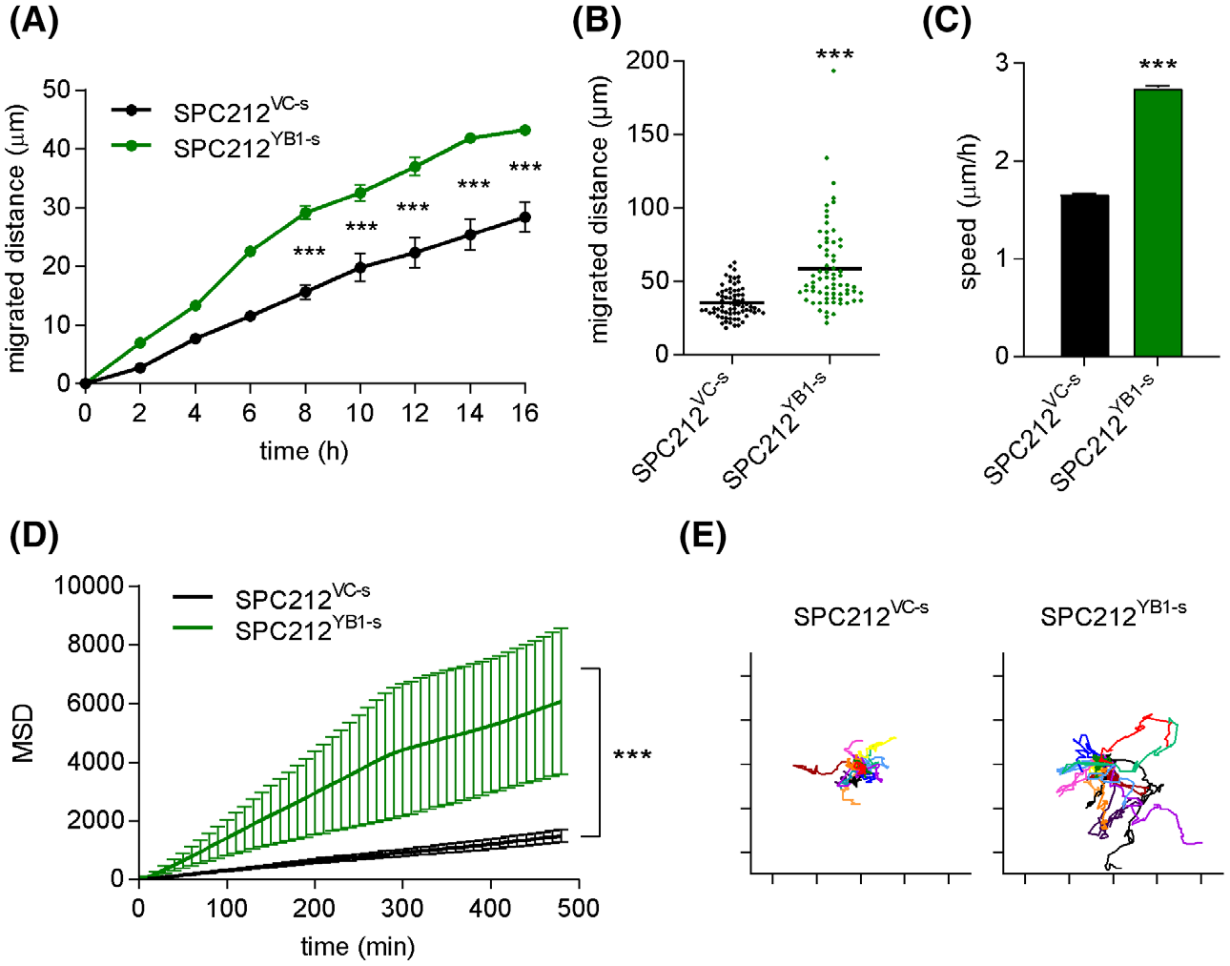
YB‐1 overexpression increases cell migration. (A) Migrated distance of SPC212 cells stably overexpressing YB‐1 (SPC212^YB1‐s^) or the empty vector (SPC212^VC‐s^) in wound healing assays. Data is shown as mean ± SEM of three biological replicates. (B) Cumulative migrated distance of single cells over 24 h, quantified by manual single cell tracking. Each dot represents one single cell. The horizontal lines indicate the mean. (C) Average speed, (D) mean squared displacement (MSD) and (E) origin plots of tracked cells (*N* = 70) were calculated by DiPer and are shown as mean ± SEM. Student's *T*‐test (with Holm‐Sidak correction in A), ***P < 0.001.

Since scattering and elongated cell shape are associated with cell migration, we analyzed the migratory behavior of SPC212 cells using a wound healing assay. YB‐1 overexpression significantly decreased time taken for gap closure (Fig. 2A). To further understand the effect of YB‐1 overexpression on migration, we conducted single cell tracking on SPC212^YB1‐s^ and SPC212^VC‐s^ cells. YB‐1 overexpressing cells displayed an average increase in total migrated distance and migration speed (Fig. 2B,C). Additionally, the mean squared displacement (MSD), a parameter for the directionality of the movement, was also significantly increased (Fig. 2D), visualized in origin plots, showing representative tracks of 10–15 single cells relative to one origin (Fig. 2E). This data suggest that increasing YB‐1 protein levels causes cells to migrate further, at higher speeds and in more of the surrounding space, facilitating a migratory phenotype.

### 3.3 YB‐1 overexpression induces steps of metastasis *in vitro* and *in vivo*

**Fig. 3:**
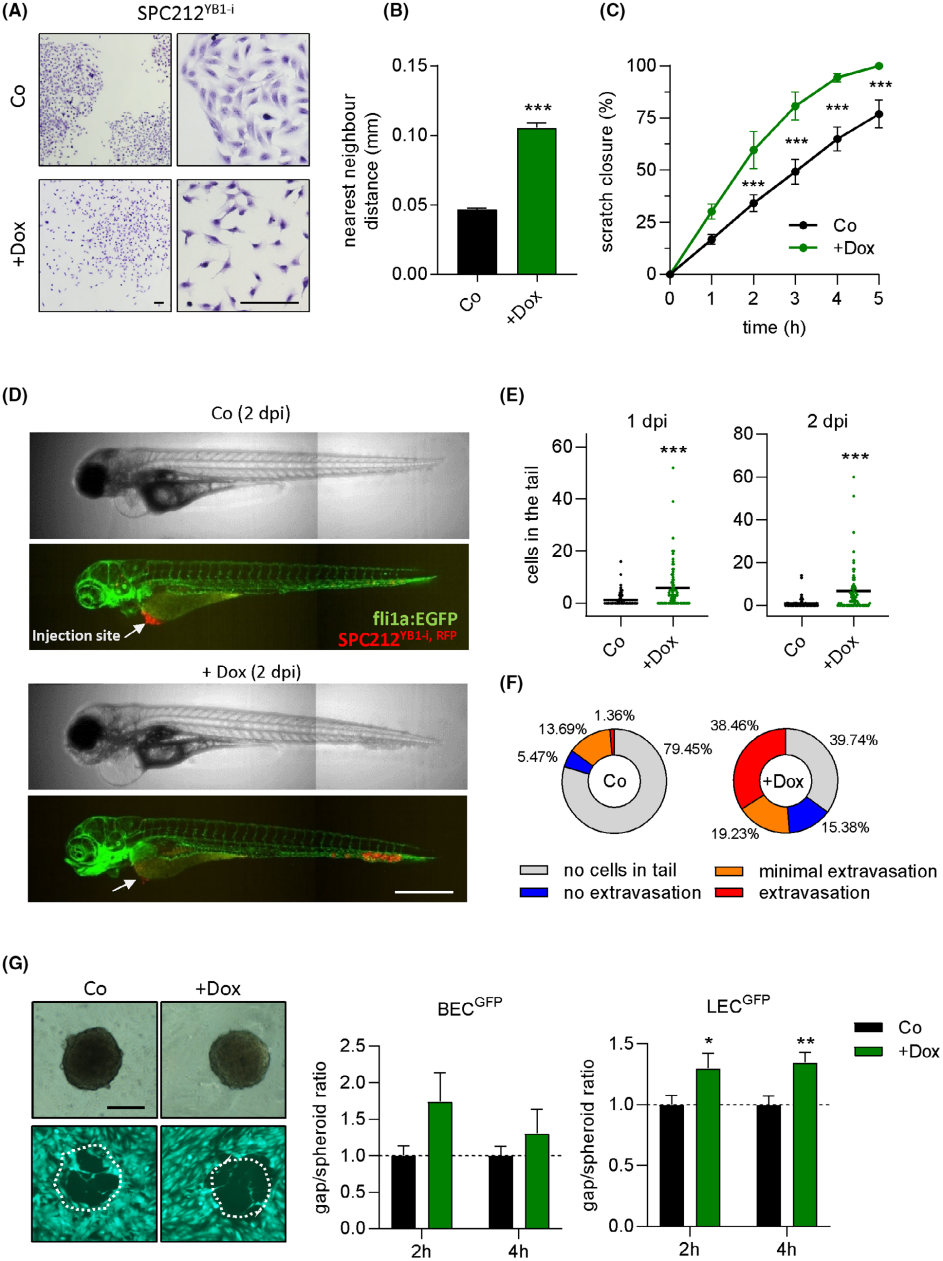
Doxycycline‐inducible YB‐1 overexpression increases cell migration. (A) Representative pictures of SPC212^YB1‐i^ cells in a colony formation assay, with or without 100 ng·mL^−1^ doxycycline (dox, Co). Scale bar: 100 μm. Experiments were performed twice in triplicates. (B) Distance to the nearest neighbor of > 120 cells derived from colony formation assays. Data are shown as mean ± SEM. (C) Percentage of wound closure in a wound healing assay with or without 100 ng·mL^−1^ doxycycline (dox). Data are shown as mean ± SEM of 4 biological replicates. (D) Representative pictures of fli1a:EGFP transgenic zebrafish without (Co) and with 100 μg·mL^−1^ doxycycline (+dox) 2 days post injection (dpi) with SPC212^YB1‐I,RFP^ cells. The injection site is indicated by the white arrow. Scale bar: 500 μm. Experiments were performed twice. (E) Quantification of tumor cells present in the tail treated as indicated after 1 and 2 dpi. Each dot represents one fish. The horizontal lines indicate the mean. (F) Percentage of fish (*N* = 73 for Co and *N* = 78 for dox) with no cells (gray), no extravascular cells (blue), < 20% extravascular cells (orange) and > 20% extravascular cells (red) in the tail at 2 dpi. (G) Representative pictures of SPC212^YB1‐i^ spheroids and GFP‐expressing LEC^GFP^ cells and quantification (mean ± SEM) of the spheroid/gap ratio with 100 ng·mL^−1^ doxycycline (+dox) in relation to the normalized control (Co) after 2 and 4 h. The dashed line marks the value of the normalized control. 10–15 spheroids per group were analyzed. The white dotted circles in the pictures represent the outline of the respective spheroid. Scale bar: 200 μm. Student's *T*‐test (with Holm‐Sidak correction in C), *P < 0.05, **P < 0.01, ***P < 0.001.

To rule out the possibility that the effects seen with the stable overexpression system result from clonal effects or adaptive changes, we decided to use a second overexpression method. To that aim, we generated SPC212 cells with doxycycline‐inducible YB‐1 expression using a retroviral construct (SPC212^YB1‐i^) and achieved YB‐1 overexpression on mRNA (3.59‐fold), and protein (1.76‐fold) levels in response to doxycycline treatment that were comparable to the stable expression system which resulted in 2.33‐ and 1.52‐fold mRNA and protein overexpression, respectively (Fig. S2). Moreover, doxycycline treatment of SPC212^YB1‐i^ resulted in effects very similar to stable overexpression of YB‐1 with respect to cell scattering, morphology changes, and migration (Fig. 3A–C). Doxycycline treatment of cells with inducible overexpression of RFP instead of YB‐1, in contrast, had no effect on YB‐1 expression or cell behavior (Fig. S3).

To study the impact of YB‐1 overexpression also *in vivo*, we performed cell motility experiments in a zebrafish model. Doxycycline‐inducible YB‐1 overexpressing cells also stably expressing RFP (SPC212^YB1‐i, RFP^) were xenotransplanted into the perivitelline space of 48 h old zebrafish larvae and their migration was followed over 2 days with or without doxycycline. Analysis of pictures taken 1 and 2 dpi revealed a highly significant increase in cells that had migrated into the tail in the doxycycline‐treated group (Fig. 3D,E). YB‐1 overexpression in the tumor cells comparable to the *in vitro* results was confirmed by qPCR in total RNA isolated from the fish using human‐specific Taqman probes (Fig. S4). In order to investigate whether the tumor cells were able to infiltrate the surrounding tissue, we quantified the amount of extravascular cells after 2 dpi and found that, indeed, the majority of fish in the doxycycline group showed > 20% extravascular cells (Fig. 3F, Fig. S5). To rule out that doxycycline treatment alone has an effect on tumor cell migration in this model, we also tested SPC212 cells stably expressing mCherry from a doxycycline‐independent CMV promoter (SPC212^mCherry^) and found no effect of doxycycline treatment (Fig. S6).

Next, since the cell migration observed in our zebrafish model also indicates extravasation out of—as well as intravasation into—vessels, we conducted a previously described [21] co‐culture intravasation assay, where tumor cell spheroids were placed on a confluent layer of GFP‐expressing blood‐ (BEC^GFP^) or lymphatic endothelial cells (LEC^GFP^) and CCID (circular chemorepellent‐induced defect) formation was observed under the microscope. Indeed, we found enhanced CCID formation after 2 and 4 h in lymphatic endothelial cell layers, when tumor spheroids were pretreated with doxycycline, whereas a similar trend in blood endothethial layers did not reach significance (Fig. 3G).

### 3.4 Combined knockdown and inducible expression of YB‐1 allows bidirectional control and rescue of cell migration

Encouraged by our findings in SPC212^YB1‐i^ cells, we generated 5 additional PM cell lines overexpressing YB‐1 in a doxycycline‐inducible manner, achieving a 1.7‐ to 4‐fold YB‐1 overexpression on mRNA, and 1.17 to 6.05‐fold on protein level (Fig. S7). All of them showed a significant increase of cell migration in response to doxycycline as observed with SPC212^YB1‐i^ (see below), as well as increased CCID formation (Fig. S8).

In a previous study, we found that YB‐1 knockdown using siRNA decreases cell migration [10]. To further substantiate the role of YB‐1 in PM cell migration, we set up an experiment enabling bidirectional control and rescue of YB‐1 expression using doxycycline‐inducible expression of YB‐1 and two siRNAs. One binds in the coding region of YB‐1 (si‐YB1^CDS^) and thus targets endogenous as well as doxycycline‐induced YB‐1 while the other binds in the 3′UTR of YB‐1 (si‐YB1^UTR^) and, therefore, only targets endogenous but not doxycycline‐induced YB‐1. Both siRNAs and the nonsilencing control siRNA have been previously validated [10, 13].

**Fig. 4:**
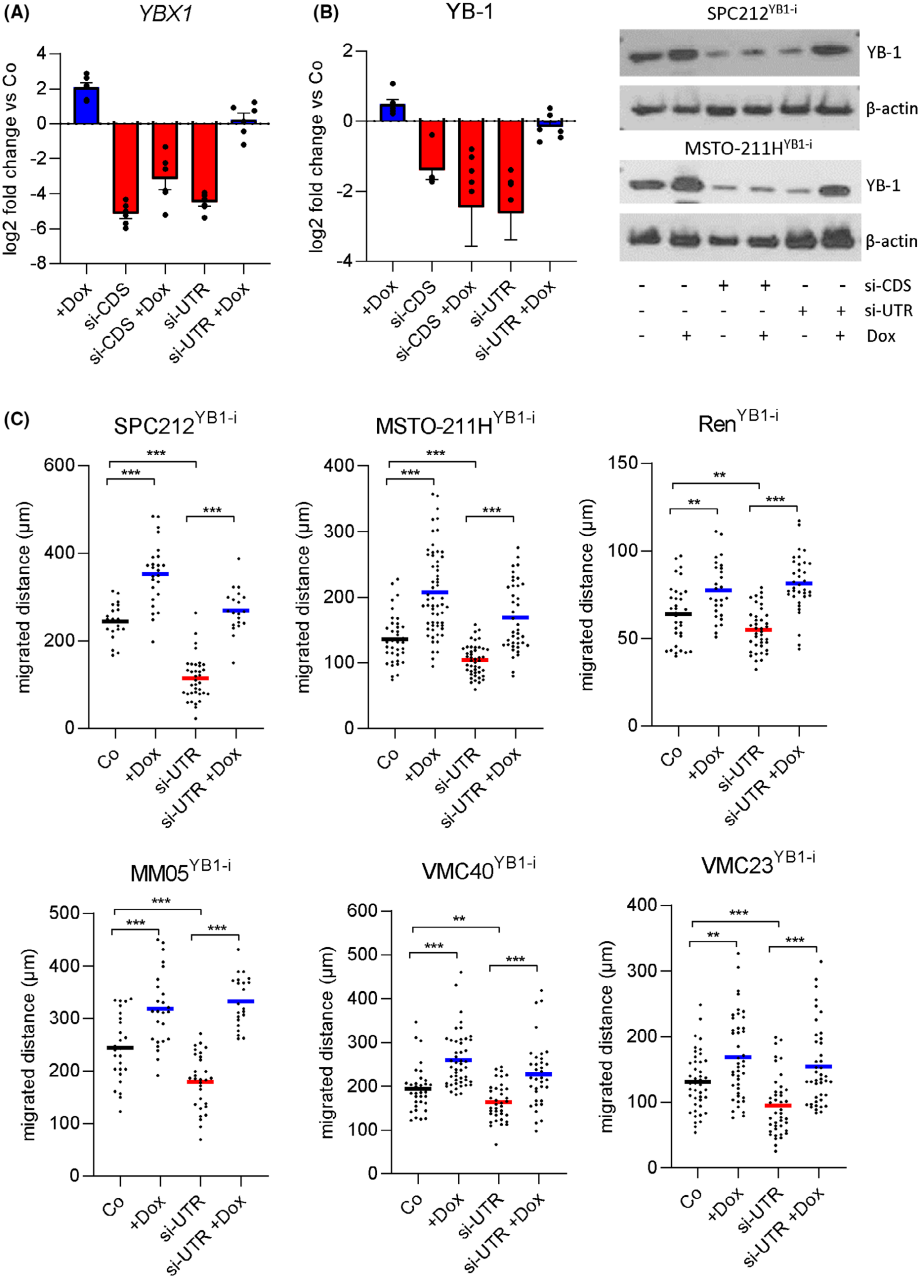
YB‐1 levels directly affect PM cell migration. Cells were transfected with 5 nM of si‐YB1^CDS^ (si‐CDS), si‐YB1^UTR^ (si‐UTR) or control siRNA (Co) and on the next day treated with 100 ng·mL^−1^ doxycycline (+dox). (A) Log2 fold change of *YBX1* mRNA levels after 48 h. Each dot represents the mean of one cell line, derived from 3 biological replicates performed in duplicates. Data are shown as mean ± SEM. (B) Representative pictures and YB‐1 protein levels relative to control‐siRNA‐transfected cells (Co) derived from densitometric analysis of western blots (*N* = 3). Data are shown as mean ± SEM. (C) Cumulative migrated distance of single cells after siRNA transfection and treatment with doxycycline (+dox) as indicated within 72 h. Quantification was performed using manual single cell tracking in imagej. Each dot represents one cell and the horizontal lines indicate the mean. ANOVA and Tukey's multiple comparisons test. **P < 0.01, ***P < 0.001.

As expected, addition of doxycycline alone induced YB‐1 mRNA and protein overexpression, while both siRNAs reduced the levels of YB‐1 across all inducible cell lines (Fig. 4A,B, Fig. S9). Meanwhile, when doxycycline was added to cells treated with the coding region‐targeting siRNA si‐YB1^CDS^, YB‐1 levels decreased (Fig. 4A,B, Fig. S9). However, when doxycycline was added to cells transfected with the 3′UTR‐targeting si‐YB1^UTR^, YB‐1 expression was comparable to normal (Fig. 4A,B, Fig. S9). This confirmed the validity of the bidirectional YB‐1 modulation system in these cell lines.

Following the above validation data, we investigated whether the effect on PM cell migration followed the expression pattern trends in this system. Doxycycline alone significantly increased the migrated distance of all six inducible cell lines, while transfection with the 3′UTR‐targeting si‐YB1^UTR^ alone resulted in significantly decreased migrated distances (Fig. 4C). Fittingly, the addition of doxycycline to YB‐1 siRNA‐transfected cells rescued the antimigratory effects of YB‐1 knockdown and in some cases increased cell migration when si‐YB1^UTR^ was used (Fig. 4C). This data strongly suggest that YB‐1 is a critical player in PM cell migration.

Epithelioid and biphasic PM, which are represented by our cell line panel, account for the big majority of PM cases. To confirm that similar mechanisms are also relevant for sarcomatoid PM and normal mesothelium, we tested the effects of YB‐1 knockdown on cell motility in the nonmalignant mesothelial cell line Met‐5A and 2 cell lines established from sarcomatoid PM (Meso62, Meso84). Migration analysis also showed a significant reduction of cell migration with both YBX1‐targeting siRNAs (Figs S10 and S11).

### 3.5 YB‐1 levels affect the expression of EGFR, snail, MMP1, EPHA5, and PARK2

Having established a system for bidirectional modulation of YB‐1 expression in 6 cell lines, we aimed to identify downstream targets of YB‐1 that may mediate its control of mesothelioma cell migration. We selected a panel of stem cell markers (*NANOG*, *OCT4*, *SOX2*) and migration‐related genes (*ZEB1*, *SNAI1*, *SNAI2*, *TWIST1*, *VIM*, *EGFR*, *MMP1*, *MMP2*), which have been previously described to be regulated by YB‐1 [5, 9, 22, 23, 24, 25]. Furthermore, we added the ephrin receptor tyrosine kinase *EPHA5*, the ubiquitin ligase parkin (*PARK2*), the carboxypeptidase A4 (*CPA4)* and the negative mTOR regulator *DDIT4*. These four genes were selected based on our previous RNAseq analysis of the mesothelioma cell lines MSTO‐211H, VMC23 and Ren transfected with si‐YB1^CDS^ [13]. While expression of *EPHA5 and PARK2* was strongly upregulated, the one of *CPA4 and DDIT4* was reduced in all three cell lines (Fig. S12).

**Fig. 5:**
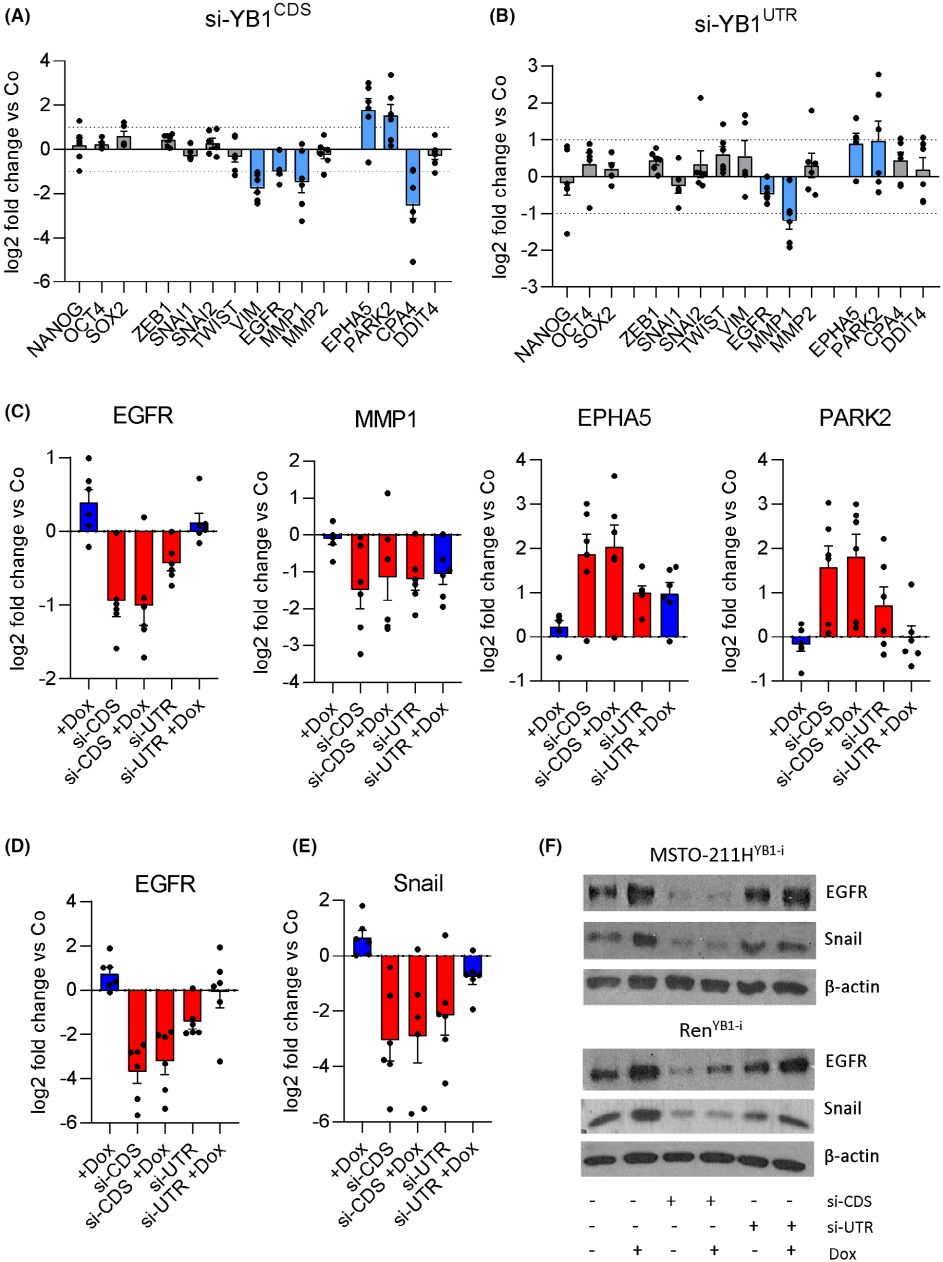
YB‐1 regulates the expression of EGFR and snail. Expression levels of target gene mRNA 48 h after transfection with 5 nm of (A) si‐YB1^CDS^ and (B) si‐YB1^UTR^, relative to control siRNA transfected cells (Co). Each dot represents the mean of one cell line, derived from three biological replicates performed in duplicates. Data are shown as mean ± SEM. Blue bars indicate a mean log2 fold change > 1. (C) Expression levels of target gene mRNA 48 h after transfection with 5 nm of si‐YB1^CDS^ (si‐CDS) or si‐YB1^UTR^ (si‐UTR) and treatment with 100 ng·mL^−1^ doxycycline (+dox), relative to control siRNA‐transfected cells (Co). Each dot represents the mean of one cell line, derived from three biological replicates performed in duplicates. Data are shown as mean ± SEM. (D) EGFR and (E) snail protein levels relative to control‐siRNA‐transfected cells (Co) derived from densitometric analysis of western blots (*N* = 3), 48 h after transfection with 5 nm siRNA and treatment with or without 100 ng·mL^−1^ doxycycline at the time of transfection as indicated. Each dot represents the mean of one cell line. Data is shown as mean ± SEM. (F) Representative pictures of the western blot.

First, we transfected the cells with si‐YB1^CDS^ and determined the mean expression of the target genes across all six cell lines. When setting a threshold of mean log2 fold change > 1 across the whole panel of cell lines, 9 of the 15 selected target genes were not consistently changed after YB‐1 knockdown, including the stem cell markers *NANOG*, *OCT4*, and *SOX2* as well as *ZEB1*, *SNAI1*, *SNAI2*, *TWIST1*, *MMP2*, and *DDIT4* (Fig. 5A). In contrast, *MMP1*, *EGFR*, *VIM*, and *CPA4* were widely downregulated, while *EPHA5* and *PARK2* were upregulated (Fig. 5A). We then validated these results using si‐YB1^UTR^. Generally, the expression changes in the target genes were similar but less pronounced, except for *VIM* and *CPA4*, which were not regulated in the same direction by si‐YB1^CDS^ and si‐YB1^UTR^ (Fig. 5B). In summary, we found that the transcript levels of *EGFR and MMP1 were downregulated*, *while EPHA5* and *PARK2* were upregulated in response to YB‐1 knockdown using both siRNAs in mesothelioma cells.

We have previously shown that EGFR and MMP1 act as key players in PM motility and EMT (epithelial to mesenchymal transition)‐like changes [26], and EPHA5 and PARK2 have been described to both drive and inhibit tumor cell migration [27, 28, 29, 30]. Therefore, we evaluated these genes in the doxycycline‐inducible YB‐1 overexpression and rescue model by qPCR. We found that EGFR expression follows the direction of YB‐1 levels in both directions in the majority of cell lines tested (Fig. 5C). *MMP1*, *EPHA5*, and *PARK2* were only partially affected by YB‐1 overexpression on the mRNA level (Fig. 5C) and can thus not account for the stimulation of cell migration by YB‐1. Regulation of EGFR expression by YB‐1 was confirmed on the protein level by immunoblotting and largely reflected the mRNA results (Fig. 5D,F). Additionally, despite not being regulated at the mRNA level, snail was included in the immunoblots because it was previously described to be translationally regulated by YB‐1 and is known as a regulator of EMT [9]. Indeed, similar to EGFR, snail showed a close posttranscriptional coregulation by YB‐1 in the majority of PM cell lines (Fig. 5E,F).

### 3.6 YB‐1 regulates PM cell migration via snail

**Fig. 6:**
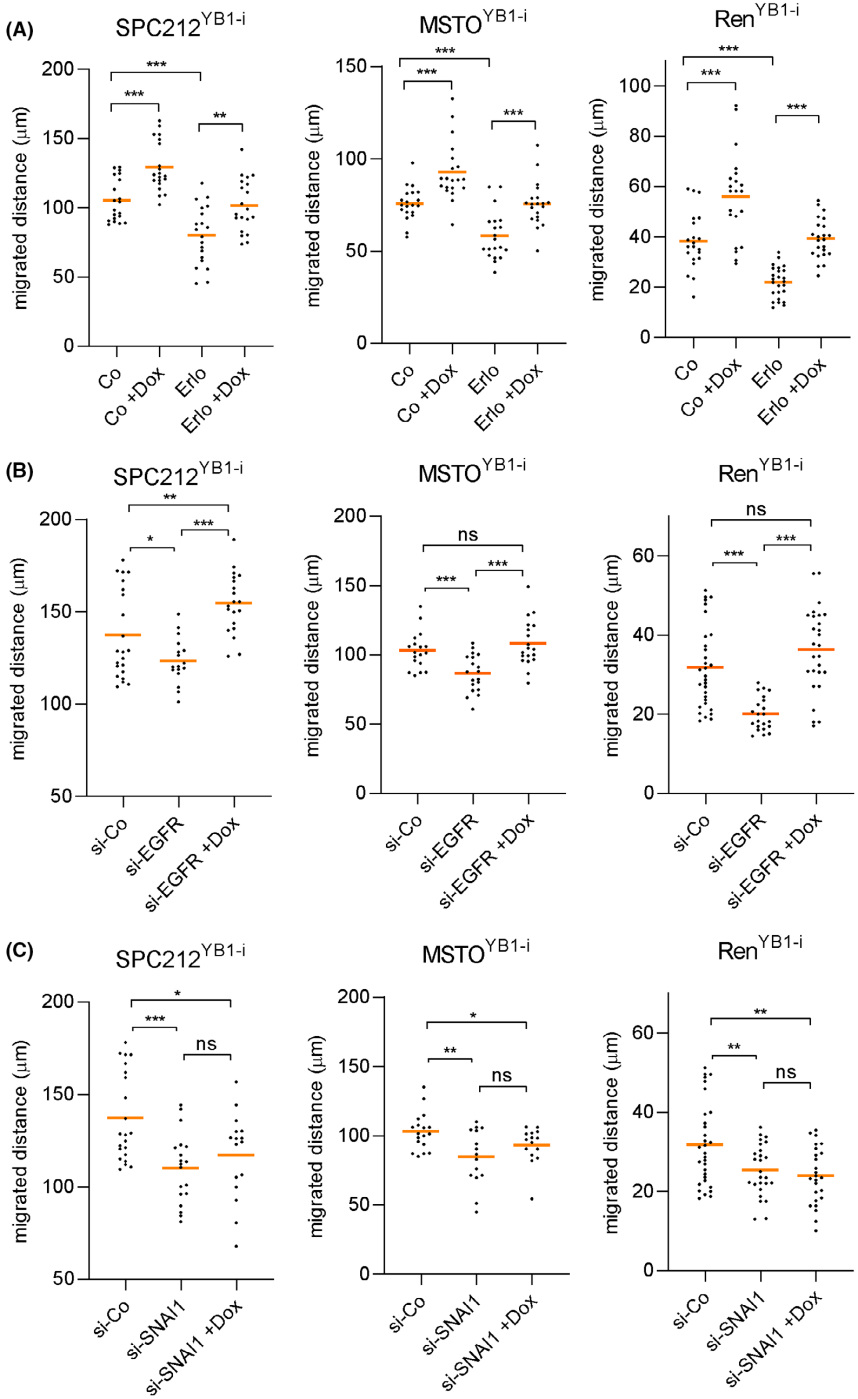
Snail but not EGFR inhibition prevents YB‐1‐induced PM cell migration. (A) Cells were exposed to 10 μm erlotinib (Erlo) or DMSO (Co) and after 24 h treated with 100 ng·mL^−1^ doxycycline (+dox) as indicated. Cells were transfected with 10 nm (15 nm for Ren^YB1‐i^) of (B) EGFR‐specific (si‐EGFR), (C) SNAI1‐specific (si‐SNAI1) or control (si‐Co) siRNA and on the next day treated with 100 ng·mL^−1^ doxycycline. Cumulative migrated distance was measured via live cell videomicroscopy for 48 h. Experiments were performed two times in quadruplicates. Quantification was performed using manual single cell tracking of > 20 cells per group in imagej. Each dot represents one cell, and the horizontal lines indicate the mean. ANOVA and Tukey's multiple comparisons test. *P < 0.05, **P < 0.01, ***P < 0.001, ns, not significant.

Finally, to test whether EGFR and/or snail are required for YB‐1‐induced cell migration, we transfected three cell lines, MSTO‐211H^YB1‐i^, SPC212^YB1‐i^ and Ren^YB1‐I^, with siRNAs targeting EGFR or snail (Fig. S13) and monitored their migratory behavior with and without doxycycline. Additionally, erlotinib was used to pharmaceutically inhibit the EGFR function. While both knockdown and pharmaceutical inhibition of EGFR reduced cell migration, this effect could be rescued by doxycycline‐induced YB‐1 overexpression (Fig. 6A,B). Knockdown of snail, however, resulted in no change in cell migration when YB‐1 was overexpressed (Fig. 6C) suggesting that its presence is required for YB‐1‐induced stimulation of migration in mesothelioma cells.

## 4 Discussion

Local spreading and invasion, for which cell migration is a prerequisite, are major hurdles to the development of more successful therapy in PM. Identifying and understanding the mechanisms that drive PM cell motility could provide new avenues to block cell migration and lead to the development of new therapeutic approaches to impair tumor spreading. Multiple signaling proteins have been shown to impact PM migration. These include, for instance, the receptor tyrosine kinase (RTK) family member AXL [31], the RTK family ligands FGF2 and EGF [26], the TGFβ family members activin A and B [32, 33] and the sheddase ADAM10 [34]. Here we demonstrate that YB‐1 is a key player in PM cell motility, which is in line with its role in several other malignancies including melanoma [35], sarcoma [36] and lung adenocarcinoma [37]. Moreover, we show that in addition to stimulating growth and migration *in vitro*, YB‐1 overexpression also stimulates intravasation in a 3D co‐culture model and cell spreading in a zebrafish model. While the former has, to the best of our knowledge, not been demonstrated for YB‐1 in any tumor type before, the latter is in agreement with data in sarcoma, where YB‐1 was shown to promote metastasis via translational activation of hypoxia inducible factor (HIF1α) [36].

PM cells generally express rather high levels of YB‐1, which stems at least in part from the loss of microRNA miR‐137 that targets YB‐1 [10]. The degree of protein overexpression resulting from either the stable or inducible ectopic expression system was below 10‐fold compared to endogenous levels in all cases, suggesting that the observed effects are unlikely to be caused by unrealistically high expression levels. Treatment with siRNAs resulted in a similar degree (< 10‐fold) of downregulation of YB‐1 protein, which is also comparable to results previously achieved with a miR‐137 mimic [5]. Both approaches together allowed us to investigate known and novel putative target genes of YB‐1 in PM with respect to their degree of dependency on YB‐1 levels and their relevance in the control of PM cell migration. Multiple targets of YB‐1 have been described in a variety of normal and malignant cell types and we focused on those previously connected to cell migration [5, 6]. We recently published an ingenuity pathway analysis from RNAseq data of MSTO‐211H, VMC23 and Ren cells transfected with YB‐1 si‐YB1^CDS^ [13]. Besides regulation of cell cycle‐related pathways, these data indicated a strong to moderate association with cell function terms including “Migration,” “Invasion,” “Movement,” and “Cell–Cell Contact” as well as “Rho GTPase Signaling” and “Upstream Kinase EGFR” in all three cell lines. From the differentially expressed genes in the RNAseq data, our current study confirms regulation of *EPHA5* and *PARK2* mRNA in response to YB‐1 knockdown in several additional PM cell lines. Although it is not yet clear whether these genes are direct or indirect targets and their regulation on the protein level still needs to be demonstrated, they could represent interesting candidates for further investigation in the manifold functions of YB‐1. Overall, EGFR, which was previously shown to be transcriptionally regulated by YB‐1 in breast cancer cells [7, 8], showed an excellent fit in expression levels with YB‐1. Based on this, it is likely that YB‐1 is directly involved in EGFR transcription in PM. Additionally, EGFR itself was previously shown to induce enhanced migration and EMT in PM cells [26] and multiple other cell types [38]. In lung adenocarcinoma, EGFR was linked to increased migration, but not EMT [15]. Indeed, knockdown or pharmacological inhibition of EGFR also reduced migration in our study, suggesting ligand‐dependent or ‐independent activation of EGFR in these cells. Since whole‐genome gene expression microarray and RNAseq data (https://www.ebi.ac.uk/arrayexpress accession number E‐MTAB‐8986, Gene Expression Omnibus data repository accession number GSE153368) suggest that PM cells express several EGFR ligands, ligand‐mediated activation of EGFR is likely. Nevertheless, YB‐1‐induced stimulation of PM cell migration could clearly proceed despite impaired EGFR expression and in presence of an EGFR inhibitor, demonstrating that an EGFR‐independent pathway mediates YB‐1‐induced stimulation of migration.

To investigate this alternative pathway, we investigated the relationship between YB‐1 and snail, the zinc finger transcription factor encoded by the SNAI1 gene, in PM cells. Snail represses the cell adhesion molecule E‐cadherin and thus is involved in cell adhesion, migration, and EMT in multiple cell types [39, 40]. In PM, snail was shown to be a negative prognostic marker [41] and linked to cisplatin resistance [42]. Our data show that in PM cells, modulation of YB‐1 levels alters snail protein but not mRNA in the same direction. This is consistent with a study in breast epithelial cells, which demonstrated that snail was translationally controlled by YB‐1 [9]. Furthermore, snail knockdown significantly reduced PM cell migration and importantly blocked YB‐1 induced stimulation. This strongly suggests that the YB‐1‐driven invasive phenotype in PM cells is dependent on pathways involving YB‐1 translational control of snail. Snail is a classic marker of EMT [43], but EMT‐independent stimulation of cell survival and motility has also been reported [44]. In PM, EMT was previously linked to more sarcomatoid features and increased aggressiveness [26, 45]. Interestingly, however, inhibition of cell motility by YB‐1 silencing was observed in cell lines from all main histological subtypes and in nonmalignant mesothelial cells, suggesting that presence of an EMT phenotype is not a strict prerequisite for the inhibition of migration.

While snail protein expression was significantly associated with shorter survival of PM patients [41], such data are not yet available for YB‐1 on the protein level. Of note, analysis of the TCGA (The Cancer Genome Atlas) transcriptomics data for mesothelioma has shown that high YB‐1 mRNA levels are significantly associated with shorter PM patient survival [5]. Future immunohistochemistry studies will clarify whether YB‐1 protein expression has prognostic implications and might serve as a stratifying marker for PM subsets.

## 5 Conclusions

Overall, our study suggests that YB‐1 is a significant contributing factor to the aggressive behavior of PM and, specifically, its function of post‐transcriptionally regulating snail appears to be critical for its role in promoting PM motility.

## Conflict of interest

All authors declare that they have no known competing financial interests or personal relationships that could have appeared to influence the work reported in this paper.

## Author contributions

KS: Conceptualization, data curation, formal analysis, funding acquisition, investigation, methodology, project administration, supervision, validation, visualization, writing—original draft, writing—review and editing. SE, BZ, MP, TGJ, DE, AW, HP, CS, AR: Formal analysis, investigation, methodology, writing—review and editing. KH, MAH, WB, BD: Resources, supervision, validation, writing—review and editing. MD, GR: Conceptualization, funding acquisition, methodology, resources, supervision, writing—review and editing. MG: Conceptualization, funding acquisition, project administration, resources, supervision, validation, visualization, writing—original draft, writing—review and editing.

### Peer review

The peer review history for this article is available at https://publons.com/publon/10.1002/1878‐0261.13367.

## Supporting information


**Fig. S1.** Stable overexpression of YB‐1 in SPC212.
**Fig. S2.** Induction of YB‐1 overexpression by doxycycline in SPC212 cells.
**Fig. S3.** Cell migration after induced expression of YB‐1 and RFP.
**Fig. S4.** Induction of YB‐1 in SPC212 cells after injection into zebrafish larvae.
**Fig. S5.** Detection of extravascular cells in zebrafish tails.
**Fig. S6.** Doxycycline treatment in a doxycycline‐independent model in zebrafish larvae.
**Fig. S7.** Inducible overexpression of YB‐1 in a larger panel of PM cell lines.
**Fig. S8.** CCID (circular chemorepellent‐induced defect) formation in response to YB‐1 induction.
**Fig. S9.** YB‐1 protein levels in response to doxycycline and siRNA treatment.
**Fig. S10.** Knockdown of *YBX1* in mesothelial and sarcomatoid PM cells.
**Fig. S11.** Cell migration after YB‐1 silencing in mesothelial and sarcomatoid PM cells.
**Fig. S12.** Expression change of *EPHA5*, *PARK2*, CPA4, and *DDIT4* after YB‐1 silencing.
**Fig. S13.** Expression change of *EGFR* and *SNAI1* after siRNA treatment.
**Table S1.** Taqman probes and primers used for qPCR.

## Data Availability

The data that support the findings of this study are available in the main figures and the supplementary material of this article.
