# Supplementary material for: YB‐1 regulates mesothelioma cell migration via snail but not EGFR, MMP1, EPHA5 or PARK2
Source: Mol Oncol. 2023 Jan 25;18(4):815–31. doi: 10.1002/1878-0261.13367 (PMC10994239; doi:10.1002/1878-0261.13367)
Supplement: Supplementary file 1 — Fig. S1. Stable overexpression of YB‐1 in SPC212. Fig. S2. Induction of YB‐1 overexpression by doxycycline in SPC212 cells. Fig. S3. Cell migration after induced expression of YB‐1 and RFP. Fig. S4. Induction of YB‐1 in SPC212 cells after injection into zebrafish larvae. Fig. S5. Detection of extravascular cells in zebrafish tails. Fig. S6. Doxycycline treatment in a doxycycline‐independent model in zebrafish larvae. Fig. S7. Inducible overexpression of YB‐1 in a larger panel of PM cell lines. Fig. S8. CCID (circular chemorepellent‐induced defect) formation in response to YB‐1 induction. Fig. S9. YB‐1 protein levels in response to doxycycline and siRNA treatment. Fig. S10. Knockdown of YBX1 in mesothelial and sarcomatoid PM cells. Fig. S11. Cell migration after YB‐1 silencing in mesothelial and sarcomatoid PM cells. Fig. S12. Expression change of EPHA5, PARK2, CPA4, and DDIT4 after YB‐1 silencing. Fig. S13. Expression change of EGFR and SNAI1 after siRNA treatment. Table S1. Taqman probes and primers used for qPCR. [file MOL2-18-815-s001.pdf]

Supplementary Table S1.

Taqman probes and primers used for qPCR.

Gene name	Taqman ID / Primer sequence
**ACTB**	Hs99999903_m1
**EGFR**	Hs01076078_m1
**GAPDH**	Hs9999905_m1
**YBX1**	Hs00358903_g1
**CPA4**	For: TCTCTACCCTCATCCACATC<br>Rev: GAGACCCACCTAAAGACAAG
**DDIT4**	For: AACAGCTGCTCATTGAGG<br>Rev: GTTCAGTCGTCTCTGTCTTG
**EPHA5**	For: GGCTTGGAGTGACTCTTG<br>Ref: GGCACCATTCCGTTTACC
**GAPDH**	For: AGCTCACTGGCATGGCCTTC<br>Rev: ACGCCTGCTTCACCACCTTC
**MMP1**	For: GTGCCTGATGTGGCTCAGTT<br>Rev: ATGGTCCACATCTGCTCTTG
**MMP2**	For: GACATCAAGGGCATTCAG<br>Rev: TGCCAAGGTCAATGTCAG
**NANOG**	For: CTCCTCCCATCCCTCATAG<br>Rev: CCACCCTCCATGAGATTG
**OCT4**	For: GAGGATCACCCTGGGATATAC<br>Rev: GGTCGTTTGGCTGAATACC
**PARK2**	For: CATGCTCTTACCCACACTAC<br>Rev: CACGATCTTCCTGAGAAGTC
**SNAI1**	For: TATGCTGCCTTCCCAGGCTTG<br>Rev: ATGTGCATCTTGAGGGCACCC
**SNAI2**	For: CAAACTACAGCGAACTGGACAC<br>Rev: GGTATGACAGGCATGGAGTAAC
**SOX2**	For: TGCACAACTCGGAGATCAG<br>Rev: CTTAGCCTCGTCGATGAAC
**TWIST1**	For: CGACGAGCTGGACTCCAAGATG<br>Rev: AGACCGAGAAGGCGTAGCTG

**VIM**	For: GGCTCAGATTCAGGAACAGC<br>Rev: CTGAATCTCATCCTGCAGGC
**YBX1**	For: GGAGTTTGATGTTGTTGAAGGA<br>Rev: GTCAGGGGAGGTCTCTTCTT
**ZEB1**	For: CCAGTGGTCATGATGAAAATGGAACACC<br>Rev: CAGACTGCGTCACATGTCTTTGATCTC

(A)

(B)

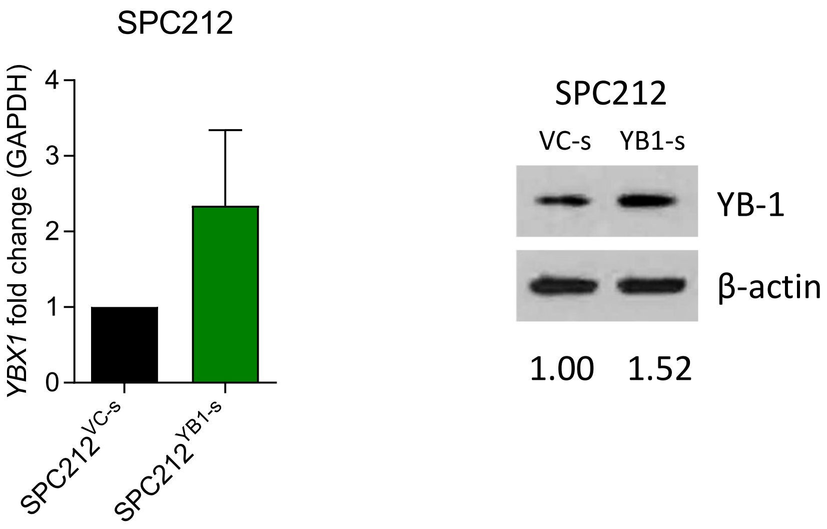

Supplementary Figure S1. Stable overexpression of YB-1 in SPC212. Stable overexpression of **(A)** *YBX1* mRNA and **(B)** YB-1 protein in SPC212$^{YB1\text{-}s}$ cells compared to SPC212$^{VC\text{-}s}$, assessed by qPCR and western blot, respectively.

Supplementary Figure S1

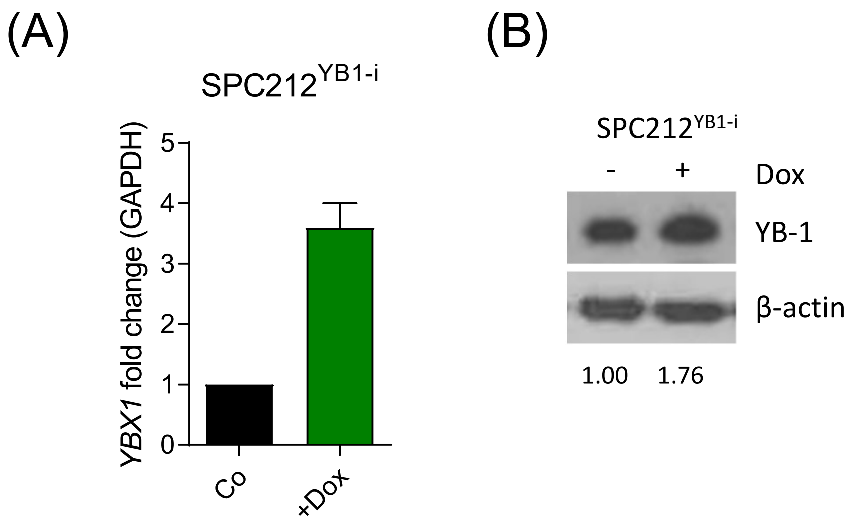

Supplementary Figure S2. Induction of YB-1 overexpression by doxycycline in SPC212 cells. Inducible overexpression of **(A)** YBX1 mRNA and **(B)** YB-1 protein in $SPC212^{YB1\text{-}i}$ cells treated with 100 ng/ml doxycycline (Dox) for 48 h compared to untreated (Co) assessed by qPCR and western blot, respectively.

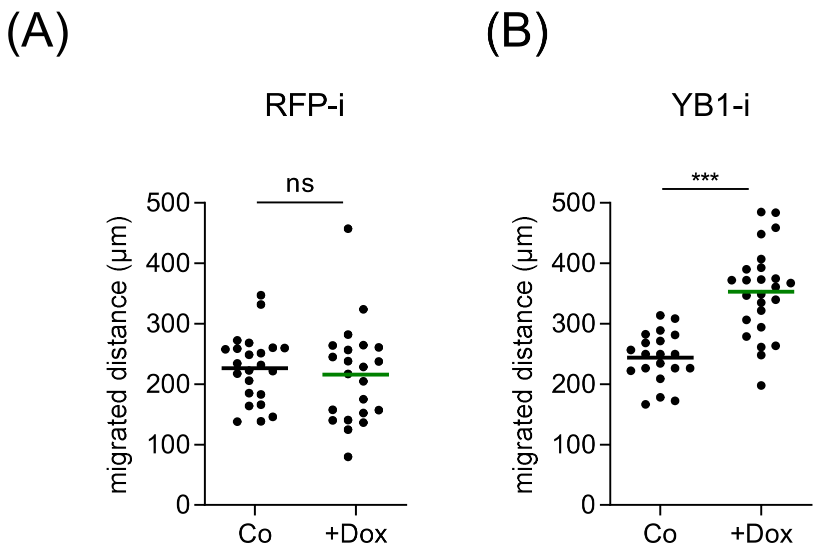

Supplementary Figure S3. Cell migration after induced expression of YB-1 and RFP. Cumulative migrated distance of single cells with doxycycline-inducible **(A)** RFP (RFP-i) or **(B)** YB-1 (YB1-i) after treatment with 100 ng/ml doxycycline (+Dox) as indicated within 72 h. Quantification was performed using manual single cell tracking in ImageJ. ***p<0.001, ns: not significant.

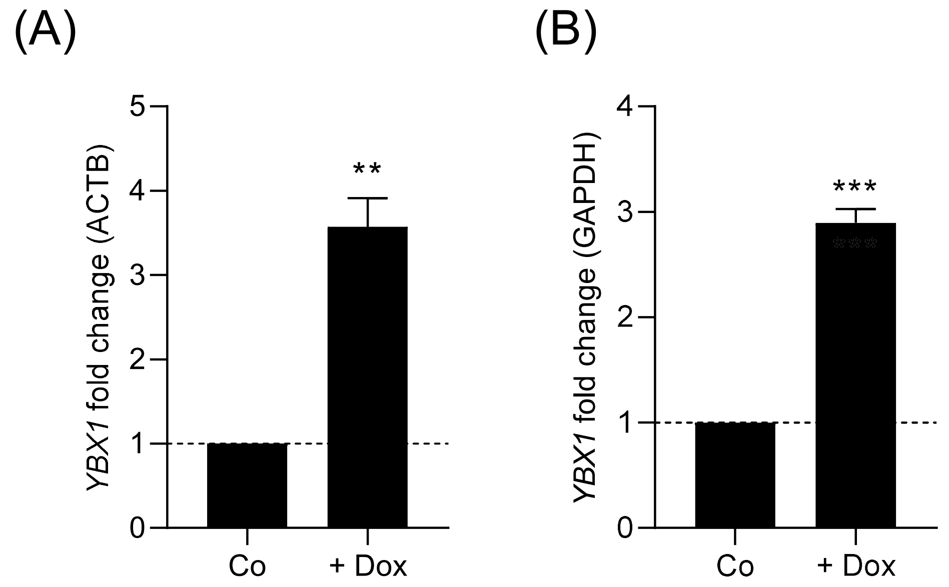

Supplementary Figure S4. Induction of YB-1 in SPC212 cells after injection into zebrafish larvae. *YBX1* mRNA levels from SPC212$^{YB1\text{-}i}$ cells injected into zebrafish larvae from the doxycycline-treated group (+Dox) compared to the untreated group (Co), relative to **(A)** β-actin and **(B)** GAPDH. RNA was isolated from pooled snap-frozen fish pellets after the last imaging (2 days post injection). For qPCR, human-specific probes were used. **p<0.01, ***p<0.001

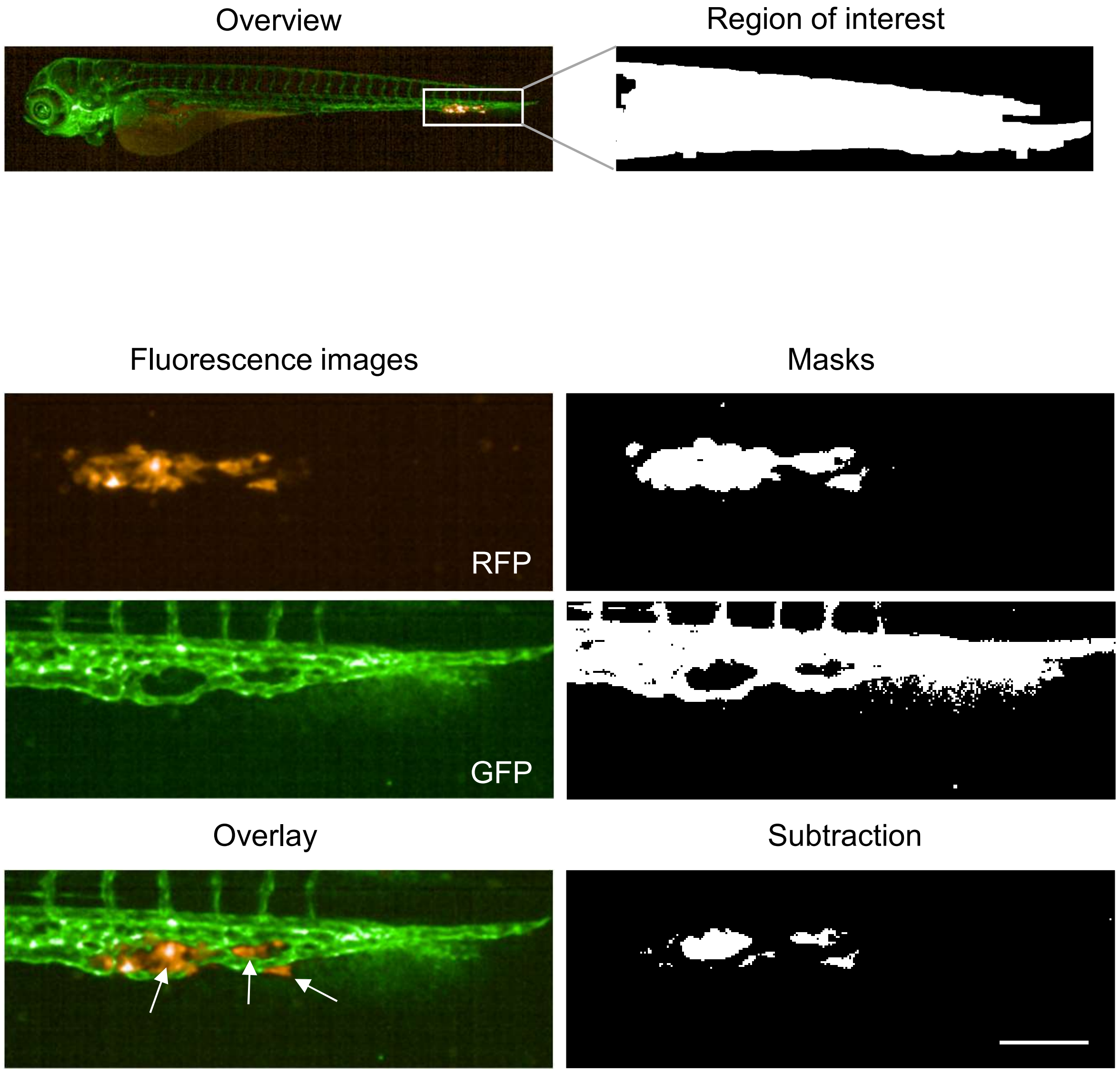

Supplementary Figure S5. Detection of extravascular cells in zebrafish tails. Representative images of the detection of extravascular cells using masks of single channels (RFP: tumor cells, GFP: vasculature) and subsequent subtraction. Arrows indicate extravascular cells. Scale bar: 100 µM

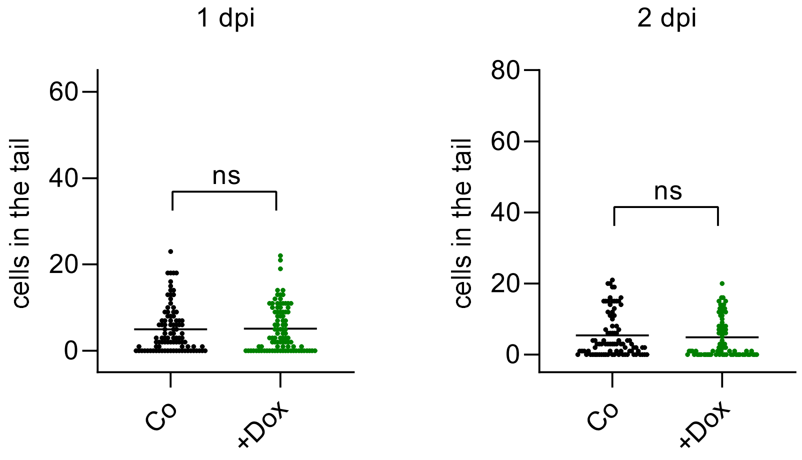

Supplementary Figure S6. Doxycycline treatment in a doxycycline-independent model in zebrafish larvae. Quantification of tumor cells present in the tail treated as indicated after 1 and 2 days post injection (dpi). Zebrafish larvae were injected with SPC212 cells expressing mCherry ($SPC212^{mCherry}$) from a doxycycline-independent promoter and divided into a control (Co) and doxycycline (+Dox, 100 µg/ml) group. Each dot represents one fish. ns: not significant

Supplementary Figure S6

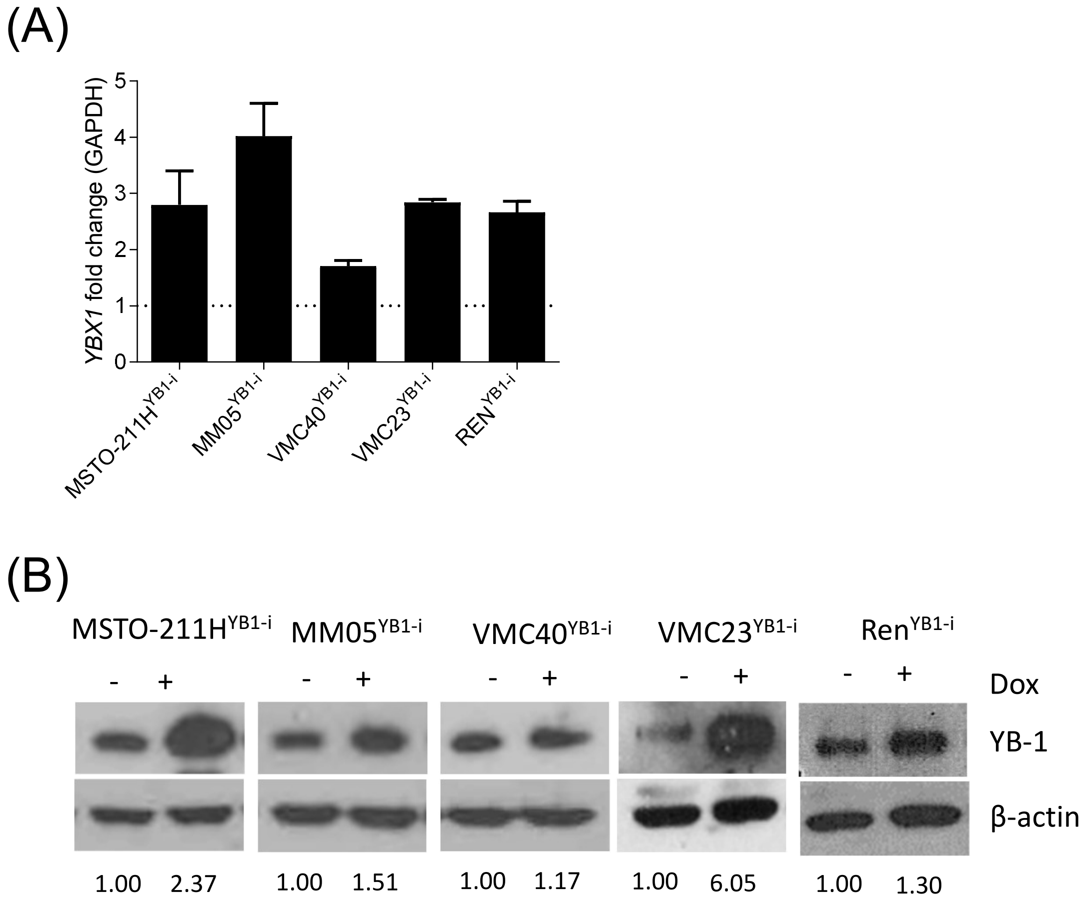

Supplementary Figure S7. Inducible overexpression of YB-1 in a larger panel of PM cell lines. Inducible overexpression in a larger panel of MPM cell lines of **(A)** *YBX1* mRNA and **(B)** YB-1 protein in cells treated with 100 ng/ml doxycycline (Dox) for 48 h, compared to untreated, assessed by qPCR and western blot, respectively.

Supplementary Figure S7

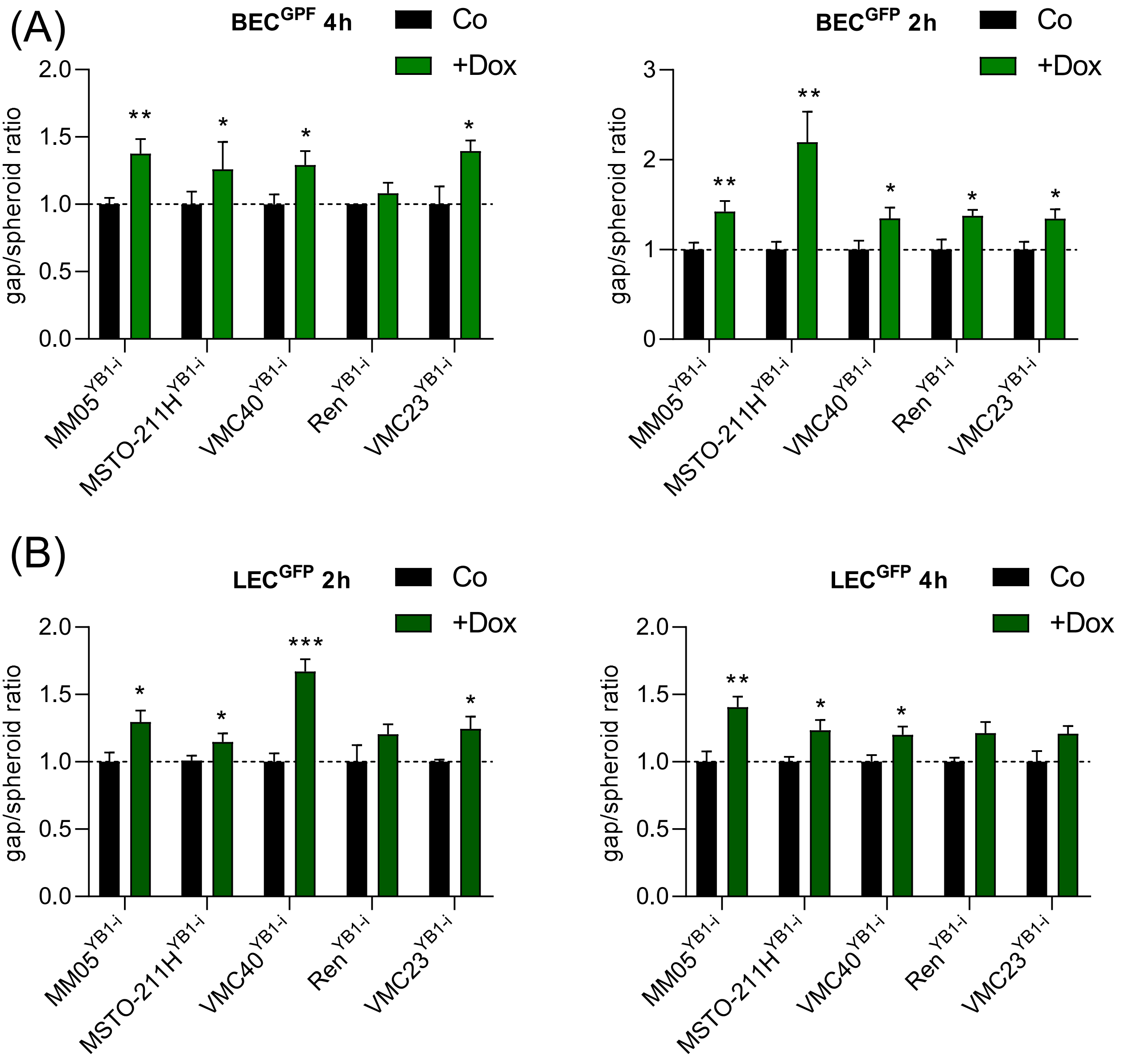

Supplementary Figure S8. CCID (circular chemorepellent-induced defect) formation in response to YB-1 induction. Gap/spheroid ratio of MPM cell co-cultures with **(A)** BEC^{GFP} or **(B)** LEC^{GFP} cells in a CCID formation assay after 2 and 4 h. Tumor cells were pre-treated with doxycycline (+Dox) or solvent (Co). *$p<0.05$, **$p<0.01$, ***$p<0.001$

Supplementary Figure S8

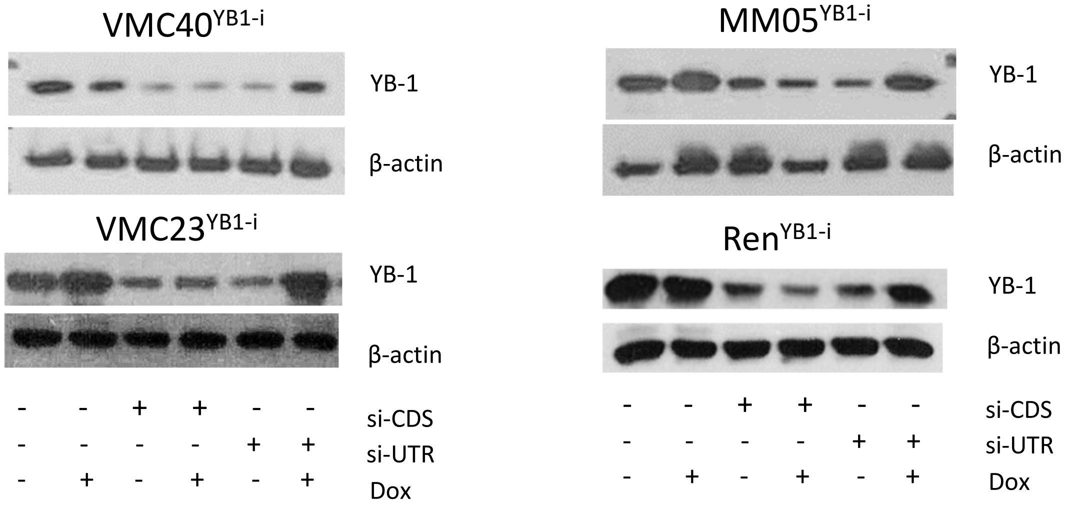

Supplementary Figure S9. YB-1 protein levels in response to doxycycline and siRNA treatment. Representative Western blot pictures of YB-1 levels relative to β-actin in doxycycline-inducible MPM cell lines transfected with 5 nM siRNA and treated with 100 ng/ml doxycycline as indicated.

Supplementary Figure S9

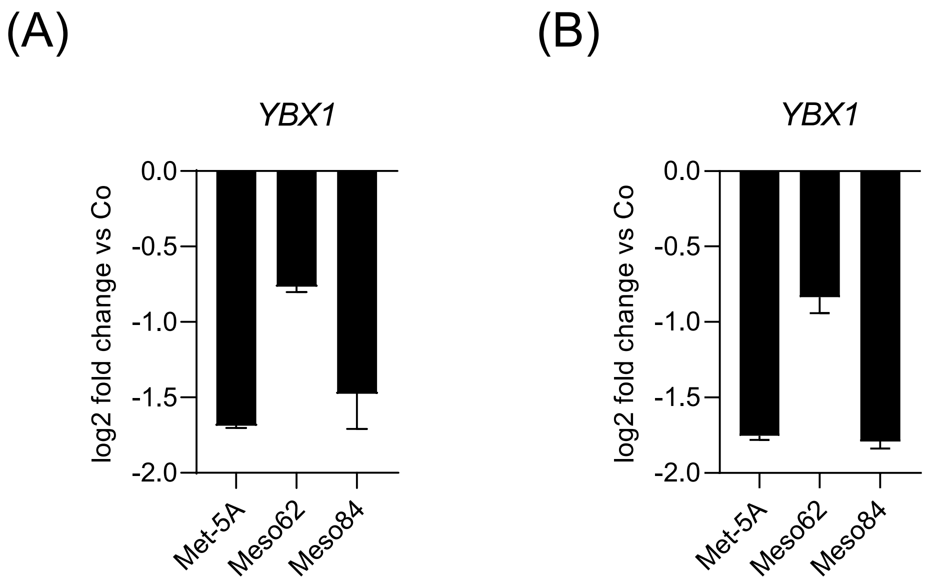

Supplementary Figure S10. Knockdown of YBX1 in mesothelial and sarcomatoid PM cells. Log2 fold expression of YBX1 in cell lines 48 hours after transfection with **(A)** 5 nM si-YB1^{CDS} and **(B)** 5 nM si-YB1^{UTR}, relative to control-siRNA (Co) transfected cells.

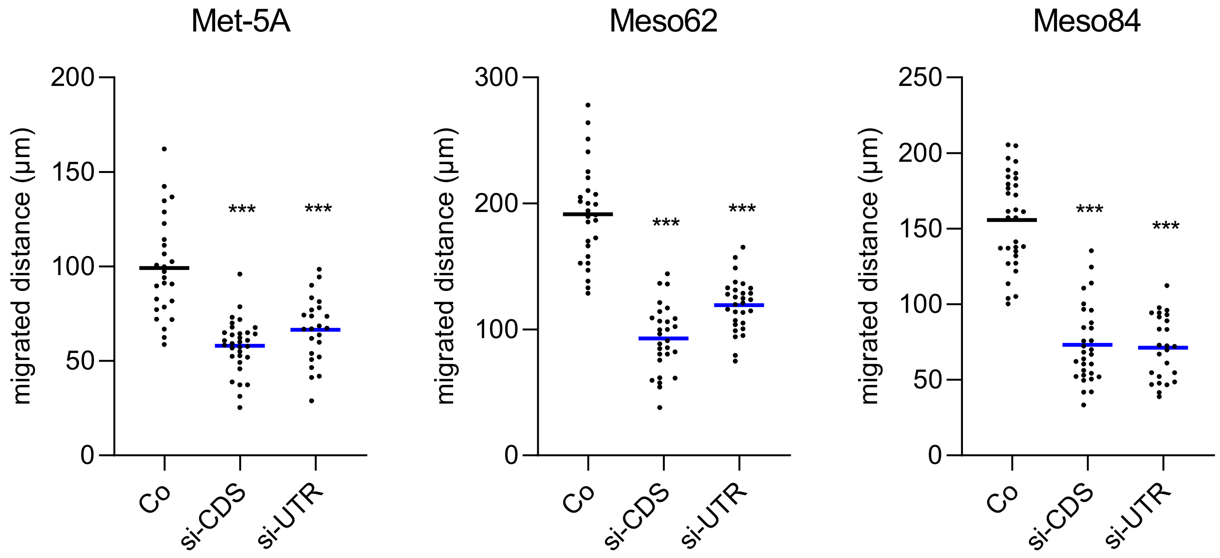

Supplementary Figure S11. Cell migration after YB-1 silencing in mesothelial and sarcomatoid PM cells. Cumulative migrated distance of normal mesothelial (Met-5A) and sarcomatoid PM (Meso62, Meso84) cells over 72 h. Cells were transfected with 5 nM si-YB1^{CDS}, si-YB1^{UTR} or control siRNA (Co) 48 h before. Quantification was performed using manual single cell tracking in ImageJ. Each dot represents one cell. ***p<0.001

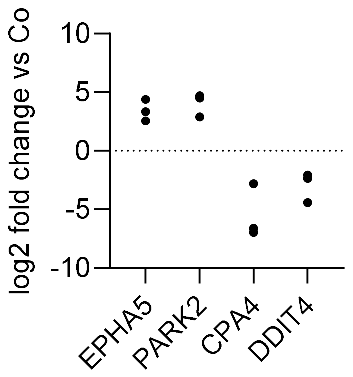

Supplementary Figure S12. Expression change of EPHA5, PARK2, CPA4 and DDIT4 after YB-1 silencing. Log2 fold change of selected genes in MSTO-211H, VMC23 and Ren cells transfected with 5 nM si-YB1^{CDS} compared to control transfected cells, derived from a previously published RNAseq analysis (Johnson et al, *Cancers* 2020). Each dot represents one cell line.

Supplementary Figure S12

(A) (B)

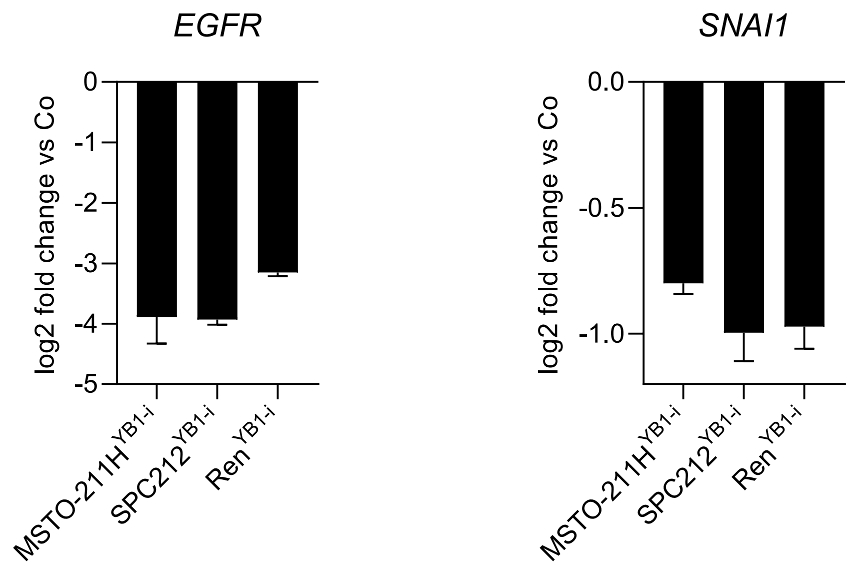

Supplementary Figure S13. Expression change of EGFR and SNAI1 after siRNA treatment. (A) Log2 fold expression of EGFR in cell lines 48 hours after transfection with 10 nM (15 nM for Ren $^{YB1\text{-}i}$) of EGFR-specific siRNA relative to control-siRNA (Co) transfected cells. **(B)** Log2 fold expression of SNAI1 in cell lines 48 hours after transfection with 10 nM (15 nM for Ren $^{YB1\text{-}i}$) of SNAI1-specific siRNA relative to control siRNA (Co) transfected cells.
